# Letter to the editor: Epidemiology of the SARS-CoV-2 variant Omicron BA.2 – vigilance needed

**DOI:** 10.2807/1560-7917.ES.2022.27.13.2200254

**Published:** 2022-03-31

**Authors:** Jing Huang, Guangting Zeng

**Affiliations:** 1 Jianghua Center for Disease Control and Prevention, Yongzhou, China; 2 The First People’s Hospital of Chenzhou, University of South China, Chenzhou, China

**Keywords:** SARS-CoV-2, COVID-19, variant of concern, Omicron, BA.2, BA.1

**To the editor: **Fonager et al. report the molecular epidemiology of the severe acute respiratory syndrome coronavirus 2 (SARS-CoV-2) variant of concern Omicron (Phylogenetic Assignment of Named Global Outbreak (Pango) lineage designation B.1.1.529) BA.2 sub-lineage in Denmark [1]. This study found significant differences in the mutation analysis of BA.1 and BA.2, but no epidemiological or clinical differences between individuals infected with BA.1 and BA.2. In their study, the hospitalisation and mortality rates of Omicron BA.1 vs BA.2 indicate that BA.2 leads to an equally mild course of coronavirus disease (COVID-19) as BA.1 compared with the Delta variant (Pango lineage designation B.1.617.2), but we cannot let our guard down on this new SARS-CoV-2 variant just yet.

This study is valuable in answering some important questions about the Omicron BA.2 variant. However, there are a few points about the study that should be treated with caution. On the one hand, the population included in this study is mainly young adults (ca 31–32 years), who had better immune protection and were less prone to hospitalisation or death, leading to an underestimation of the severity of Omicron BA.2 infection. It is well known that older adults and immunocompromised patients have low autoimmunity and generally lower neutralising antibody responses to COVID-19 vaccines. Moreover, they appear to be at greater risk of breakthrough infections and progression to hospitalisation or death at any given time after vaccination. On the other hand, it cannot be overlooked that Denmark has a high vaccination rate, which has a great impact on preventing infection and reducing the risk of hospitalisation and death. Denmark's death rate from COVID-19 has been far lower than the global average because of high vaccination rates and medical standards. The Omicron variant could still be a major health threat in low-income countries with low vaccination rates or inadequate medical resources.

In addition to these observations, cell culture experiments in human nasal epithelial cells show that BA.2 was highly replicative and more fusogenic, and viral replication experiments in hamsters show that BA.2 was more pathogenic than BA.1 [2]. Studies have indicated that some therapeutic monoclonal antibodies have lower neutralising activity against Omicron BA.2 compared with earlier SARS-CoV-2 variant strains [3,4]. Also, some models predict that the infectivity of Omicron BA.2 is ca 1.5 times that of BA.1 and 4.2 times that of Delta, with a 30 per cent greater potential to evade existing vaccines than BA.1 [5]. As a result, some scholars expect Omicron BA.2 to be the next dominating variant.

A real-world study in Denmark revealed that Omicron BA.2 was substantially more infectious than BA.1, and that BA.2 also had immune avoidance properties, increasing the likelihood that people who were not vaccinated or received only two doses of vaccine but not a booster dose would be infected with BA.2 vs with BA.1 [6]. Another study of a small sample (n = 24) of people vaccinated with Comirnaty (BNT162b2 mRNA, BioNTech-Pfizer) demonstrates that neutralising antibody titres to BA.2 were not significantly different to BA.1 but trended 1.3–1.4 fold lower; a third dose of Comirnaty was required for induction of consistent neutralising antibody titres to BA.2, similar to BA.1 [7]. Together, these two studies suggest that a third dose is necessary to contain the Omicron BA.2 epidemic.

Currently, research on Omicron BA.2 is limited. Until more research is performed to uncover the epidemiological characteristics of this variant of concern, we should remain cautious regarding Omicron BA.2. Promotion of booster vaccination, encouragement of mask-wearing and physical distancing remain effective measures as the pandemic persists.
